# Syntheses and structures of 1-[2,2-di­chloro-1-hy­droxy-3-(4-methyl­phen­yl)-3-oxoprop­yl]urea and 1-[2,2-di­chloro-3-(4-fluoro­phen­yl)-1-hy­droxy-3-oxoprop­yl]urea

**DOI:** 10.1107/S2056989025004359

**Published:** 2025-05-20

**Authors:** Firudin I. Guseinov, Tuncer Hökelek, Ksenia A. Afanaseva, Elena V. Shuvalova, Lev M. Glukhov, Aida I. Samigullina, Alebel N. Belay

**Affiliations:** a Kosygin State University of Russia, 117997 Moscow, Russian Federation; b N. D. Zelinsky Institute of Organic Chemistry, Russian Academy of Sciences, 119991 Moscow, Russian Federation; c Hacettepe University, Department of Physics, 06800 Beytepe-Ankara, Türkiye; d Department of Chemistry, Bahir Dar University, PO Box 79, Bahir Dar, Ethiopia; University of Aberdeen, United Kingdom

**Keywords:** crystal structure, hydrogen bonds, Hirshfeld surface analysis, α,α-dihalo-β-oxo­aldehydes, urea

## Abstract

The title compounds, C_10_H_9_Cl_2_FN_2_O_3_, (**I**), and C_11_H_12_Cl_2_N_2_O_3_, (**II**), are α,α-dihalo-β-diketone urea derivatives, which contain 4-fluoro­phenyl and *p*-tolyl groups, respectively. The conformation about the C_O_—C_Cl2_—C_O_—N_u_ (O = keto, Cl2 = di­chloro, u = urea) bond is *anti* in (**I**) and *gauche* in (**II**). In the crystals of both compounds, O—H⋯O hydrogen bonds generate inversion dimers and the dimers are linked into (100) layers by N—H⋯O hydrogen bonds. The Hirshfeld surface analyses of the crystal structures indicate that the most important contributions for the crystal packings are from H⋯O/O⋯H (22.3%), H⋯H (20.9%), H⋯Cl/Cl⋯H (15.6%) and H⋯C/C⋯H (10.3%) for (I) and H⋯H (31.7%), H⋯O/O⋯H (25.1%), H⋯Cl/Cl⋯H (21.1%) and H⋯C/C⋯H (9.5%) for (II).

## Abstract 2

The title compounds, C_10_H_9_Cl_2_FN_2_O_3_, (**I**), and C_11_H_12_Cl_2_N_2_O_3_, (**II**), are α,α-dihalo-β-diketone urea derivatives, which contain 4-fluoro­phenyl and *p*-tolyl groups, respectively. The conformation about the C_O_—C_Cl2_—C_O_—N_u_ (O = keto, Cl2 = di­chloro, u = urea) bond is *anti* in (**I**) and *gauche* in (**II**). In the crystals of both compounds, O—H⋯O hydrogen bonds generate inversion dimers and the dimers are linked into (100) layers by N—H⋯O hydrogen bonds.

## 1. Chemical context

The replacement of hydrogen atoms with halogen atoms at the active methyl­ene group in β-diketones prevents keto–enol tautomerism and impacts on the reactivity of the corresponding α,α-dihalo-β- diketones (*e.g*., Guseinov *et al.*, 2006 ▸). For instance, the reaction of α,α-dihalo-β-oxo­aldehydes and their derivatives with N-nucleophilic reagents lead to hetero- or macrocyclic compounds (Guseinov *et al.*, 2024 ▸). This class of compounds can be used in the spectrophotometric determination of metal ions (Aliyev *et al.*, 2020 ▸), decoration of the secondary coordination sphere of metal complexes for catalysis (Aliyeva *et al.*, 2024 ▸), crystal growth and design (Naghiyev *et al.*, 2023 ▸) and heterogenous catalysis (Mahmudov *et al.*, 2022 ▸). In fact, the use of N-compounds has many synthetic advantages (Khalilov, 2021 ▸), such as easy modification and functionalization (Huseynov *et al.*, 2021 ▸), immobilization on solid materials through supra­molecular inter­actions (Mamedov *et al.*, 2006 ▸), and crystal engineering (Hajiyeva *et al.*, 2024 ▸).
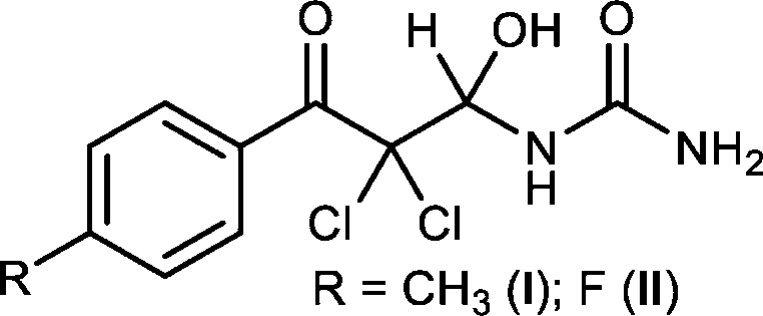


Herein, we describe the syntheses and crystal structures of the two title compounds, C_10_H_9_Cl_2_FN_2_O_3_ (**I**) and C_11_H_12_Cl_2_N_2_O_3_ (**II**), which differ in the substituent at the *para* position of the phenyl group.

## 2. Structural commentary

**Figure 1:**
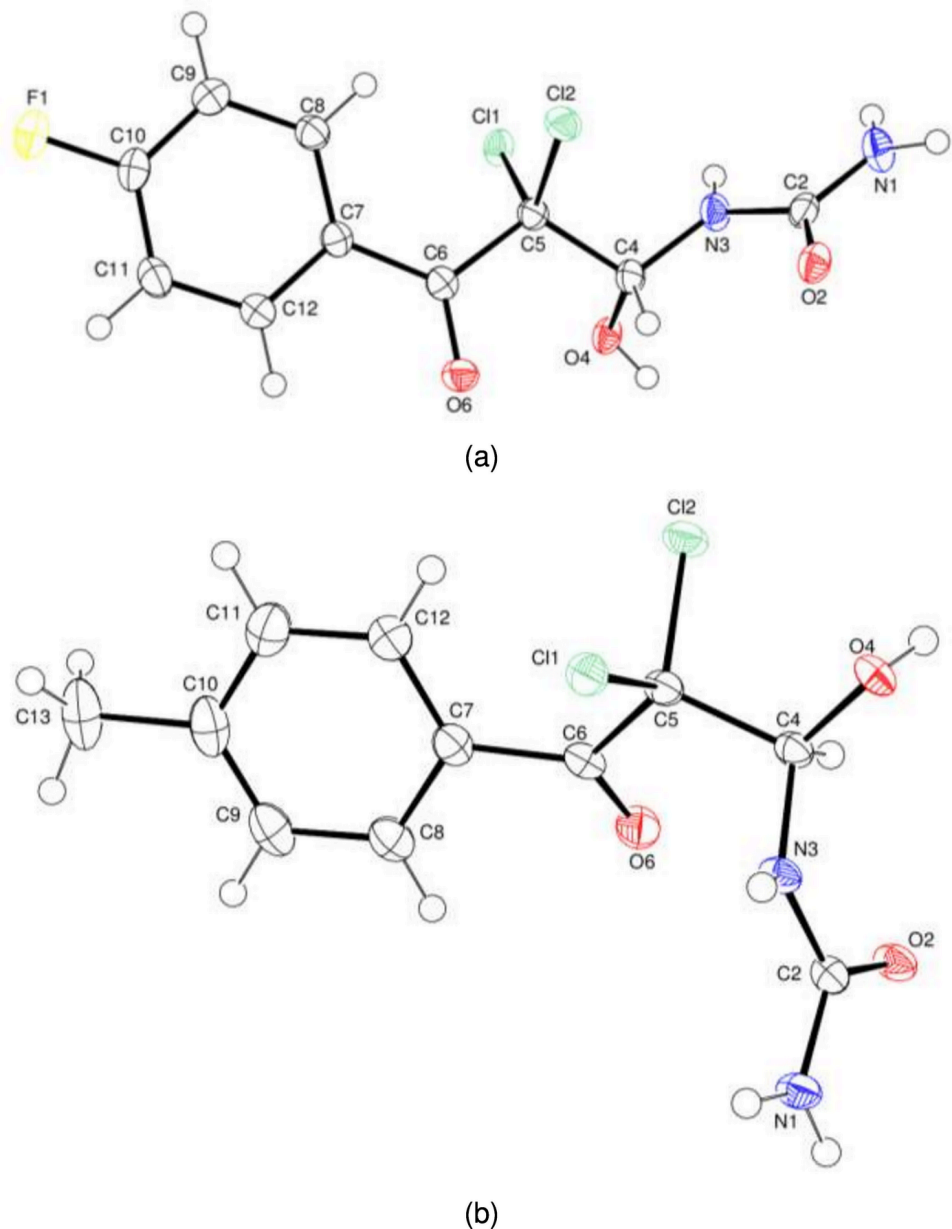
The asymmetric units of compounds (*a*) (**I**) and (*b*) (**II**) with 50% probability ellipsoids.

In (**I**) (Fig. 1 ▸), the dihedral angle between the C7–C12 phenyl group and the C2/N1/N3/O2 urea moiety is 65.19 (8)°. The key torsion angles for the backbone of the mol­ecule are C7—C6—C5—C4 = −179.65 (13), C6—C5—C4—N3 = −171.20 (12), C4—C5—C6—O6 = 2.95 (19) and C6—C5—C4—O4 = 66.65 (15)°. Atom C4 is a stereogenic centre: in the arbitrarily chosen asymmetric unit it has *R* configuration, but crystal symmetry generates a racemic mixture. Atoms F1, C6, C5 are displaced by −0.0244 (11), −0.0602 (15) and −0.0879 (15) Å, respectively, from the plane of the phenyl group. The N1—C2—O2, N3—C4—O4, N3—C4—C5, Cl1—C5—Cl2 and C4—C5—C6 bond angles in (**I**) are enlarged, while the O2—C2—N3 bond angle in (**I**) is narrowed compared to the corresponding values in (**II**): these small differences might arise due to steric reasons or ’packing effects’.

In (**II**) (Fig. 1 ▸), the corresponding dihedral angle between the C7–C12 and C2/N1/N3/O2 planes is 62.70 (9)° and the equivalent backbone torsion angles are C7—C6—C5—C4 = 162.65 (14), C6—C5—C4—N3 = −62.41 (16), C4—C5—C6—O6 = −17.42 (19) and C6—C5—C4—O4 = 176.34 (12)°. Thus it may be seen that the conformation of the atoms about the C4—C5 bond is quite different in the two structures. The stereogenic atom C4 in (**II**) was arbitrarily assigned to have an *R* configuration, but crystal symmetry generates a racemic mixture.

## 3. Supra­molecular features

**Figure 2:**
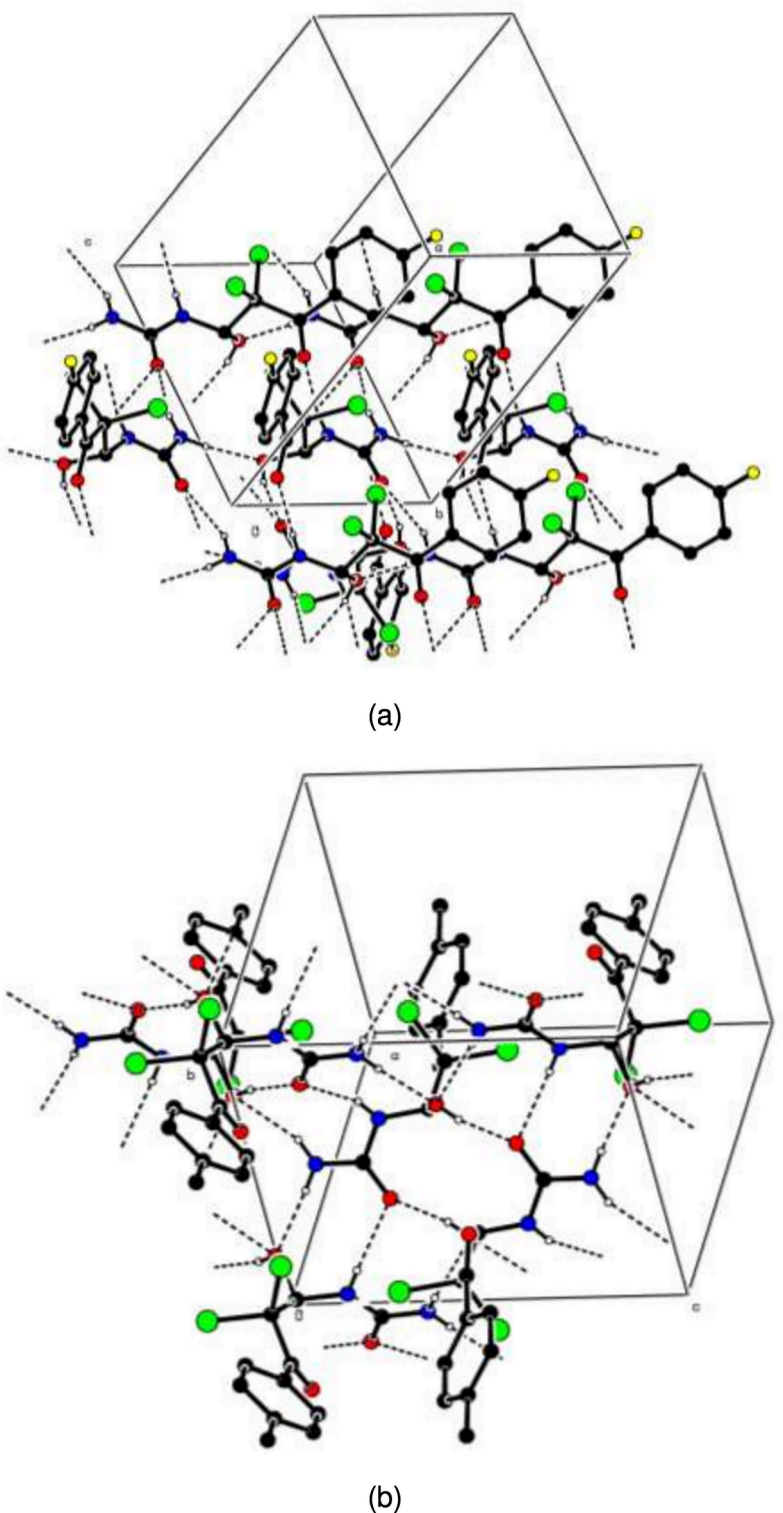
The partial packing diagrams of compounds (*a*) (**I**) and (*b*) (**II**). Inter­molecular O—H⋯O and N—H⋯O hydrogen bonds are shown as dashed lines. H atoms not involved in these inter­actions have been omitted for clarity.

In the crystals of both compounds, O—H⋯O hydrogen bonds (Tables 1 ▸ and 2 ▸) generate inversion dimers featuring 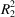
(12) loops. In both structures, N—H⋯O hydrogen bonds link the dimers into (100) layers, although they are not isostuctural. The hydrogen-bond network encloses 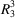
(14) loops in (**I**) (Fig. 2 ▸*a*) and 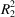
(8) and 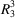
(8) loops in (**II**) (Fig. 2 ▸*b*).

## 4. Hirshfeld surface analysis

**Figure 3:**
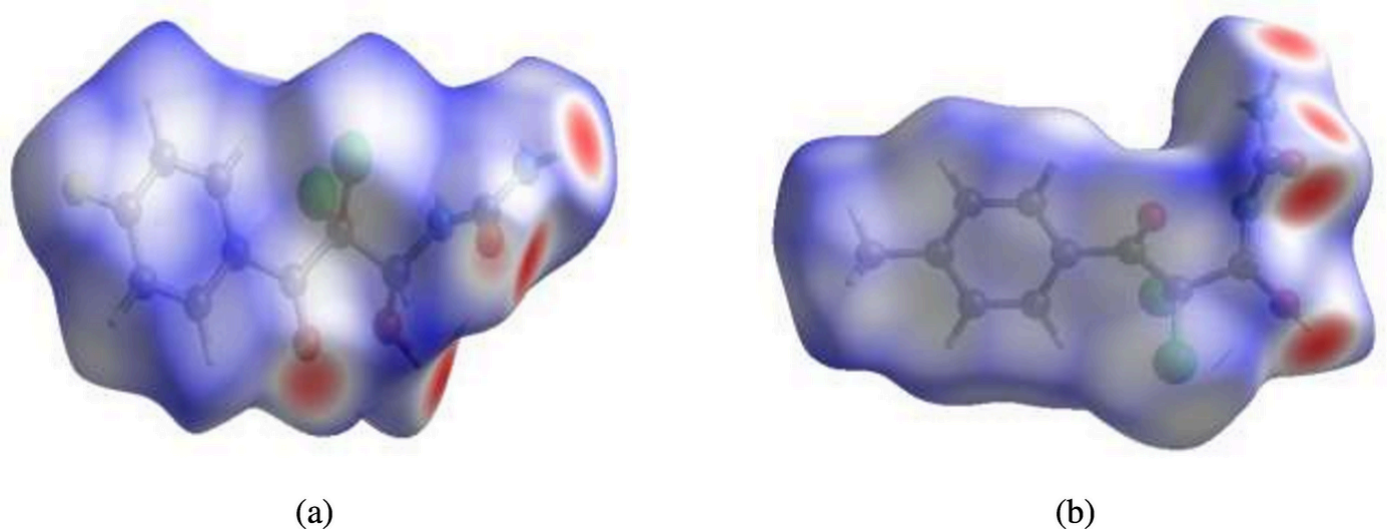
Views of the three-dimensional Hirshfeld surfaces of compounds (*a*) (**I**) and (*b*) (**II**) plotted over *d*_norm_.

**Figure 4:**
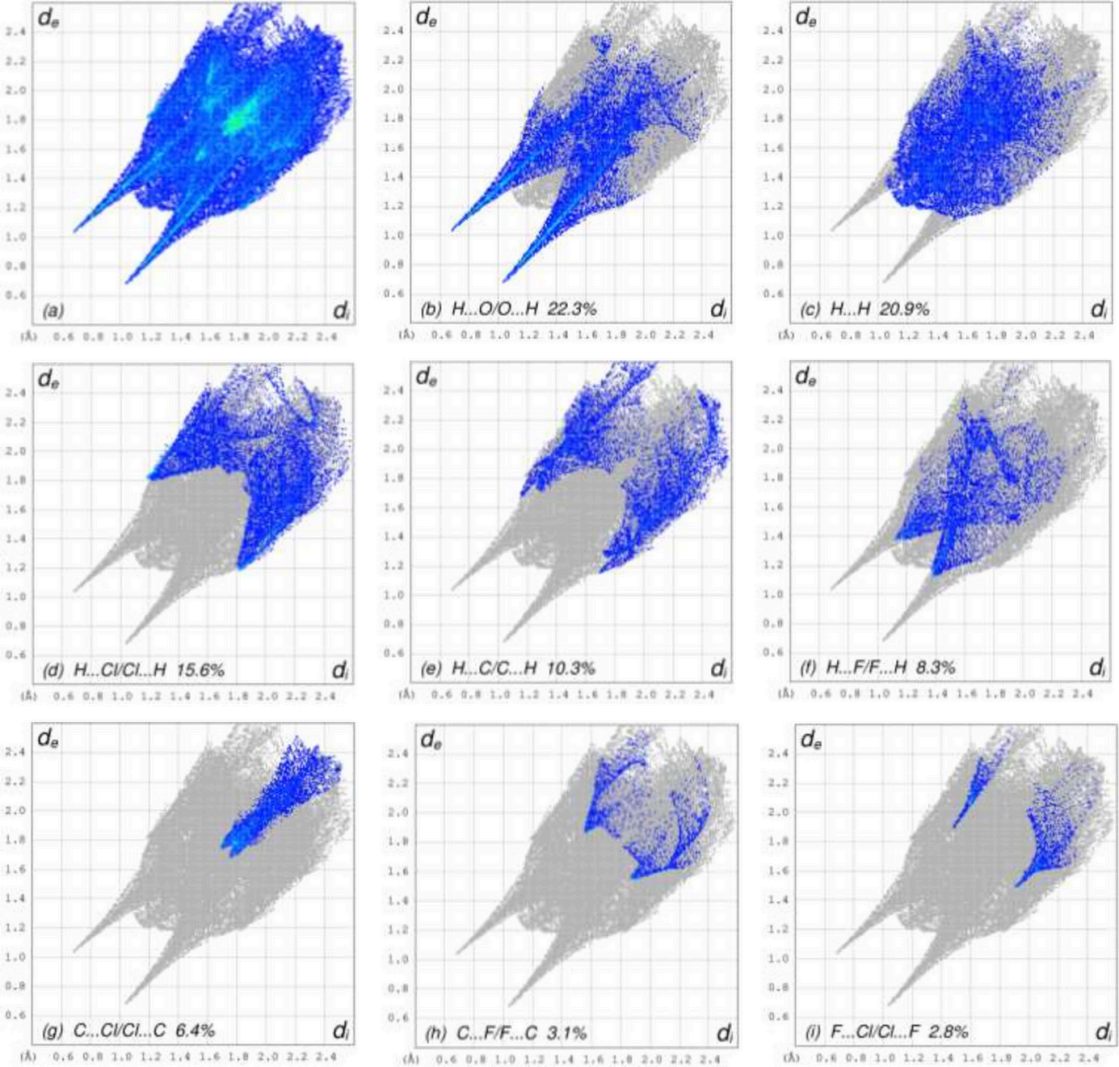
The two-dimensional fingerprint plots for compound (**I**), showing (*a*) all inter­actions, and delineated into different contact types (*b*)–(i). The *d*_i_ and *d*_e_ values are the closest inter­nal and external distances (in Å) from given points on the Hirshfeld surface.

For visualizing the inter­molecular inter­actions in the crystals of (**I**) and (**II**), Hirshfeld surface (HS) analyses were carried out using *Crystal Explorer 17.5* (Spackman *et al.*, 2021 ▸). In the HSs plotted over *d*_norm_ (Fig. 3 ▸*a* and *b*), the contact distances equal, shorter and longer with respect to the sum of van der Waals radii are shown by the white, red and blue colours, respectively. According to the two-dimensional fingerprint plots, H⋯O/O⋯H, H⋯H and H⋯Cl/Cl⋯H contacts make the most important contributions to the HSs (Table 3 ▸, Figs. 4 ▸ and 5 ▸), and they have significant differences due to the different numbers and values of the close contacts in (**I**) and (**II**).

## 5. Synthesis and crystallization

**Figure 6:**
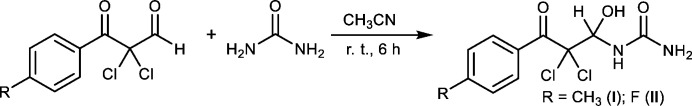
The synthesis of the title compounds.

A solution of 2,2-di­chloro-3-oxo-3-(p-tol­yl)propanal (231 mg) for (**I**) or 2,2-di­chloro-3-(4-fluoro­phen­yl)-3-oxopropanal (235 mg) for (**II**) and urea (60 mg) in 15 ml of dry aceto­nitrile was stirred at room temperature for 6 h. The solvent was removed *in vacuo*, and the remaining white powder was recrystallized from acetone solution and the title compounds were isolated. The reaction scheme is shown in Fig. 6 ▸.

(**I**): yield 82%; m.p. 378–380 K. Analysis calculated (%) for C_11_H_12_Cl_2_N_2_O_3_: C 45.38, H 4.15, N 9.62; found C 45.36, H 4.11, N 9.60. ^1^H NMR (300MHz, DMSO-*d*_6_): 2.41 (*s*, CH_3_), 5.94 (*s*, 2H, NH_2_), 6.01–6.08 (*d.d*, CH), 6.72 (*d*, OH), 6.85 (*d*. NH), 7.38 (*d*. 2H, Ar), 7.95 (*d*, 2H, Ar). ^13^C NMR (75 MHz, DMSO-*d*_6_): 21.32, 88.03, 104.05, 128.74, 128.91, 133.77, 142.81, 162.72, 186.95.

(**II**): yield 78%; m.p. 397–398 K. Analysis calculated (%) for C_10_H_9_Cl_2_FN_2_O_3_: C 40.70, H 3.07, N 9.49; found C 40.65, H 3.02, N 9.45. ^1^H NMR (300MHz, DMSO-*d*_6_): 5.96 (*s*, 2H, NH_2_), 6.03–6.08 (*d.d*, CH), 6.78 (d, OH), 6.93 (*d*, NH), 7.46 (*t*, 2H, Ar), 8.12 (*d.d*, 2H, Ar). ^13^C NMR (75 MHz, DMSO-*d*_6_): 88.10, 104.12, 115.46, 130.49, 132.37, 162.74, 167.35, 186.92.

## 6. Refinement

Crystal data, data collection and structure refinement details are summarized in Table 4 ▸. The OH and NH_2_ hydrogen atoms were located in difference-Fourier maps, and refined isotropically. The C-bond hydrogen-atom positions were placed geometrically (C—H = 0.95–1.00 Å) and refined using a riding model by applying the constraint *U*_iso_(H) = 1.2*U*_eq_(C) or 1.5*U*_eq_(methyl C).

## Supplementary Material

Crystal structure: contains datablock(s) I, II, global. DOI: 10.1107/S2056989025004359/hb8127sup1.cif

Supporting information file. DOI: 10.1107/S2056989025004359/hb8127Isup4.cml

Structure factors: contains datablock(s) I. DOI: 10.1107/S2056989025004359/hb8127Isup4.hkl

Supporting information file. DOI: 10.1107/S2056989025004359/hb8127IIsup5.cml

Structure factors: contains datablock(s) II. DOI: 10.1107/S2056989025004359/hb8127IIsup5.hkl

CCDC references: 2451169, 2451168

Additional supporting information:  crystallographic information; 3D view; checkCIF report

## Figures and Tables

**Figure 5:**
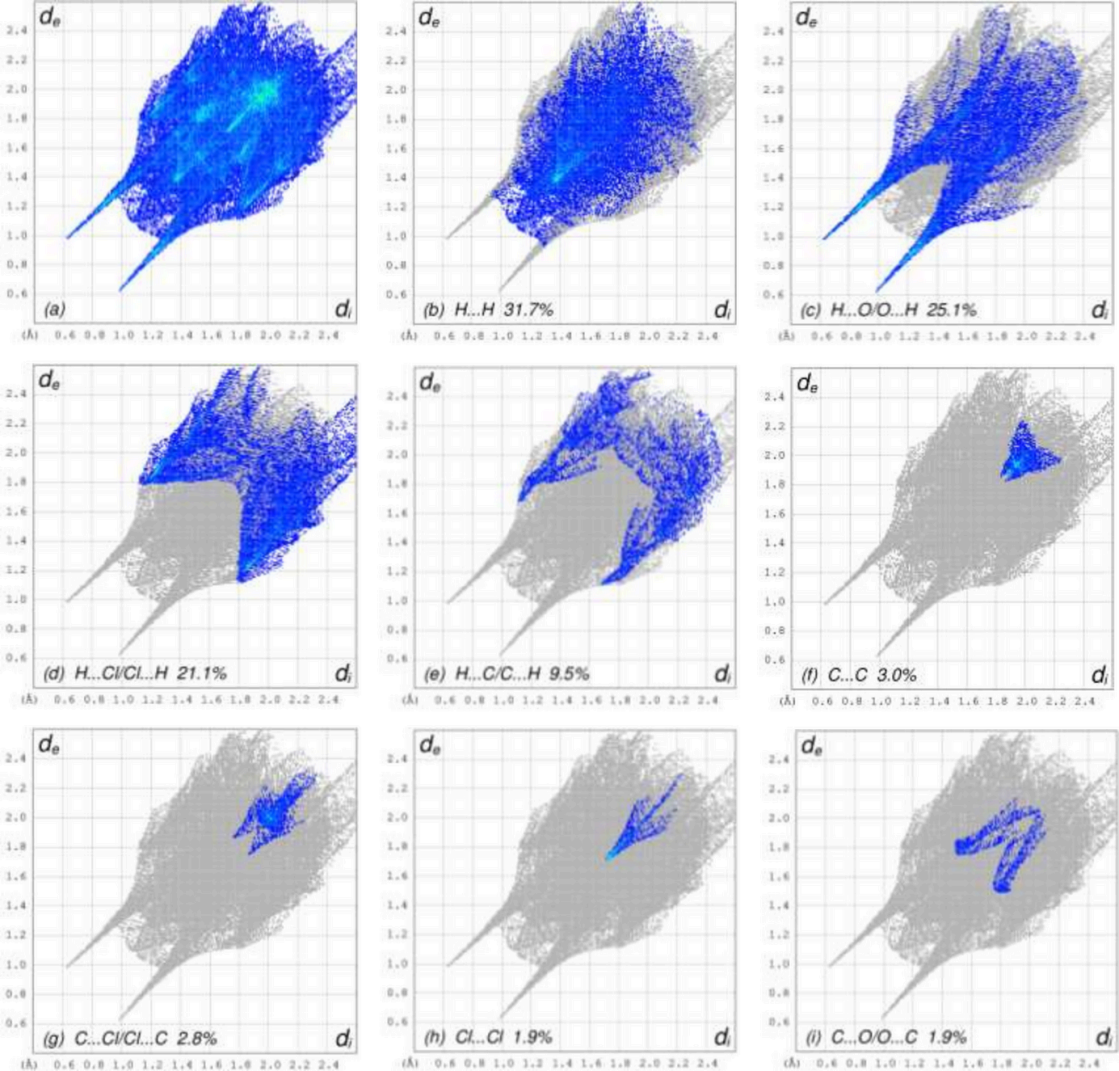
The two-dimensional fingerprint plots for compound (**II**), showing (*a*) all inter­actions, and delineated into different contact types (*b*)–(i). The d_i_ and d_e_ values are the closest inter­nal and external distances (in Å) from given points on the Hirshfeld surface.

**Table 1:** Hydrogen-bond geometry (Å, °) for (**I**)

*D*—H⋯*A*	*D*—H	H⋯*A*	*D*⋯*A*	*D*—H⋯*A*
N1—H1*A*⋯O4^i^	0.90 (3)	2.06 (3)	2.9488 (19)	172 (2)
N1—H1*B*⋯O2^ii^	0.86 (2)	2.48 (2)	3.297 (2)	158 (2)
N3—H3⋯O6^iii^	0.87 (2)	2.06 (2)	2.9055 (18)	167 (2)
O4—H4⋯O2^iv^	0.78 (3)	1.91 (3)	2.6596 (16)	161 (3)

Symmetry codes: (i) 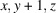; (ii) 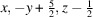; (iii) 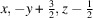; (iv) 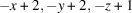.

**Table 2:** Hydrogen-bond geometry (Å, °) for (**II**)

*D*—H⋯*A*	*D*—H	H⋯*A*	*D*⋯*A*	*D*—H⋯*A*
N1—H1*A*⋯O4^i^	0.82 (3)	2.27 (3)	3.0261 (19)	153 (2)
N1—H1*B*⋯O4^ii^	0.84 (3)	2.14 (3)	2.9798 (18)	177 (2)
N3—H3⋯O2^iii^	0.81 (3)	2.19 (3)	2.9445 (18)	156 (2)
O4—H4⋯O2^iv^	0.78 (3)	1.83 (3)	2.5979 (16)	168 (3)

Symmetry codes: (i) 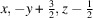; (ii) 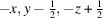; (iii) 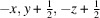; (iv) 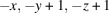.

**Table 3:** Comparison of the fingerprint percentages for compounds (**I**) and (**II**)

Contacts	**(I)**	**(II)**
H⋯O/O⋯H	22.3	25.1
H⋯H	20.9	31.7
H⋯Cl/Cl⋯H	15.6	21.1
H⋯C/C⋯H	10.3	9.5
H⋯F/F⋯H	8.3	–
C⋯Cl/Cl⋯C	6.4	2.8
C⋯F/F⋯C	3.1	–
F⋯Cl/Cl⋯F	2.8	–
Cl⋯Cl	2.4	1.9
H⋯N/N⋯H	2.4	1.3
C⋯C	1.8	3.0
O⋯O	1.4	–
F⋯F	0.7	–
O⋯Cl/Cl⋯O	0.7	0.2
C⋯O/O⋯C	0.6	1.9
N⋯O/O⋯N	0.3	0.1
C⋯N/N⋯C	0.1	–
N⋯Cl/Cl⋯N	0.1	1.3

**Table 4:** Experimental details

	(**I**)	(**II**)
Crystal data		
Chemical formula	C_10_H_9_Cl_2_FN_2_O_3_	C_11_H_12_Cl_2_N_2_O_3_
*M*_r_	295.09	291.13
Crystal system, space group	Monoclinic, *P*2_1_/*c*	Monoclinic, *P*2_1_/*c*
Temperature (K)	100	100
*a*, *b*, *c* (Å)	15.6136 (1), 7.2214 (1), 10.7929 (1)	12.62183 (13), 8.77585 (9), 11.59930 (11)
β (°)	104.622 (1)	94.8294 (9)
*V* (Å^3^)	1177.51 (2)	1280.26 (2)
*Z*	4	4
Radiation type	Cu *K*α	Cu *K*α
μ (mm^−1^)	5.14	4.60
Crystal size (mm)	0.32 × 0.15 × 0.05	0.45 × 0.35 × 0.16

Data collection		
Diffractometer	XtaLAB Synergy, Dualflex, HyPix	XtaLAB Synergy, Dualflex, HyPix
Absorption correction	Gaussian (*CrysAlis PRO*; Rigaku OD, 2024 ▸)	Gaussian (*CrysAlis PRO*; Rigaku OD, 2024 ▸)
*T*_min_, *T*_max_	0.408, 1.000	0.277, 1.000
No. of measured, independent and observed [*I* > 2σ(*I*)] reflections	16149, 2562, 2494	17344, 2808, 2738
*R*_int_	0.035	0.050
(sin θ/λ)_max_ (Å^−1^)	0.640	0.640

Refinement		
*R*[*F*^2^ > 2σ(*F*^2^)], *wR*(*F*^2^), *S*	0.031, 0.082, 1.06	0.037, 0.104, 1.06
No. of reflections	2562	2808
No. of parameters	179	180
H-atom treatment	H atoms treated by a mixture of independent and constrained refinement	H atoms treated by a mixture of independent and constrained refinement
Δρ_max_, Δρ_min_ (e Å^−3^)	0.40, −0.31	0.51, −0.43

Computer programs: *CrysAlis PRO* (Rigaku OD, 2024 ▸), *SHELXT2014/5* (Sheldrick, 2015*a* ▸), *SHELXL2018/3* (Sheldrick, 2015*b* ▸) and *OLEX2* (Dolomanov *et al.*, 2009 ▸).

## supplementary crystallographic information

### 1-[2,2-Dichloro-1-hydroxy-3-(4-methylphenyl)-3-oxopropyl]urea (I) Crystal data

**Table:**

C_10_H_9_Cl_2_FN_2_O_3_	*F*(000) = 600
*M**_r_* = 295.09	*D*_x_ = 1.665 Mg m^−^^3^
Monoclinic, *P*2_1_/*c*	Cu *K*α radiation, λ = 1.54184 Å
*a* = 15.6136 (1) Å	Cell parameters from 10775 reflections
*b* = 7.2214 (1) Å	θ = 2.9–80.5°
*c* = 10.7929 (1) Å	µ = 5.14 mm^−^^1^
β = 104.622 (1)°	*T* = 100 K
*V* = 1177.51 (2) Å^3^	Prism, colorless
*Z* = 4	0.32 × 0.15 × 0.05 mm

### 1-[2,2-Dichloro-1-hydroxy-3-(4-methylphenyl)-3-oxopropyl]urea (I) Data collection

**Table:**

XtaLAB Synergy, Dualflex, HyPix diffractometer	2562 independent reflections
Radiation source: micro-focus sealed X-ray tube, PhotonJet (Cu) X-ray Source	2494 reflections with *I* > 2σ(*I*)
Mirror monochromator	*R*_int_ = 0.035
Detector resolution: 10.0000 pixels mm^-1^	θ_max_ = 80.7°, θ_min_ = 2.9°
ω scans	*h* = −19→19
Absorption correction: gaussian (CrysAlisPro; Rigaku OD, 2024)	*k* = −9→9
*T*_min_ = 0.408, *T*_max_ = 1.000	*l* = −10→13
16149 measured reflections	

### 1-[2,2-Dichloro-1-hydroxy-3-(4-methylphenyl)-3-oxopropyl]urea (I) Refinement

**Table:**

Refinement on *F*^2^	Primary atom site location: dual
Least-squares matrix: full	Secondary atom site location: difference Fourier map
*R*[*F*^2^ > 2σ(*F*^2^)] = 0.031	Hydrogen site location: mixed
*wR*(*F*^2^) = 0.082	H atoms treated by a mixture of independent and constrained refinement
*S* = 1.06	*w* = 1/[σ^2^(*F*_o_^2^) + (0.0418*P*)^2^ + 0.7639*P*] where *P* = (*F*_o_^2^ + 2*F*_c_^2^)/3
2562 reflections	(Δ/σ)_max_ < 0.001
179 parameters	Δρ_max_ = 0.40 e Å^−^^3^
0 restraints	Δρ_min_ = −0.31 e Å^−^^3^

### 1-[2,2-Dichloro-1-hydroxy-3-(4-methylphenyl)-3-oxopropyl]urea (I) Special details

**Table:**

Geometry. All esds (except the esd in the dihedral angle between two l.s. planes) are estimated using the full covariance matrix. The cell esds are taken into account individually in the estimation of esds in distances, angles and torsion angles; correlations between esds in cell parameters are only used when they are defined by crystal symmetry. An approximate (isotropic) treatment of cell esds is used for estimating esds involving l.s. planes.

### 1-[2,2-Dichloro-1-hydroxy-3-(4-methylphenyl)-3-oxopropyl]urea (I) Fractional atomic coordinates and isotropic or equivalent isotropic displacement parameters (Å^2^)

**Table:**

	*x*	*y*	*z*	*U*_iso_*/*U*_eq_	
Cl1	0.75812 (2)	0.64065 (5)	0.20981 (3)	0.02166 (11)	
Cl2	0.71108 (2)	0.90786 (5)	0.38664 (3)	0.02237 (11)	
F1	0.45932 (7)	0.18867 (16)	0.44559 (10)	0.0321 (2)	
O2	0.91429 (7)	1.16468 (16)	0.49959 (11)	0.0231 (2)	
O4	0.93636 (8)	0.66517 (16)	0.38219 (11)	0.0216 (2)	
H4	0.9848 (18)	0.692 (4)	0.417 (2)	0.038 (7)*	
O6	0.83823 (7)	0.53628 (16)	0.55147 (10)	0.0220 (2)	
N1	0.91321 (10)	1.2742 (2)	0.30188 (16)	0.0279 (3)	
H1A	0.9206 (17)	1.389 (4)	0.334 (2)	0.040 (7)*	
H1B	0.9071 (15)	1.259 (4)	0.221 (2)	0.032 (6)*	
N3	0.88523 (9)	0.96745 (19)	0.32966 (13)	0.0222 (3)	
H3	0.8796 (15)	0.958 (4)	0.248 (2)	0.035 (6)*	
C2	0.90498 (10)	1.1385 (2)	0.38332 (16)	0.0204 (3)	
C4	0.87990 (10)	0.8058 (2)	0.40405 (14)	0.0195 (3)	
H4A	0.896975	0.839528	0.496915	0.023*	
C5	0.78496 (10)	0.7248 (2)	0.37028 (13)	0.0185 (3)	
C6	0.77450 (10)	0.5688 (2)	0.46381 (14)	0.0178 (3)	
C7	0.68898 (10)	0.4691 (2)	0.45000 (14)	0.0187 (3)	
C8	0.61114 (10)	0.5077 (2)	0.35608 (15)	0.0220 (3)	
H8	0.611405	0.600113	0.293538	0.026*	
C9	0.53355 (11)	0.4121 (2)	0.35358 (16)	0.0239 (3)	
H9	0.480694	0.437166	0.289650	0.029*	
C10	0.53522 (11)	0.2797 (2)	0.44634 (16)	0.0238 (3)	
C11	0.61052 (11)	0.2368 (2)	0.54016 (16)	0.0261 (3)	
H11	0.609462	0.144287	0.602320	0.031*	
C12	0.68768 (11)	0.3321 (2)	0.54142 (15)	0.0228 (3)	
H12	0.740343	0.304306	0.605008	0.027*	

### 1-[2,2-Dichloro-1-hydroxy-3-(4-methylphenyl)-3-oxopropyl]urea (I) Atomic displacement parameters (Å^2^)

**Table:**

	*U*^11^	*U*^22^	*U*^33^	*U*^12^	*U*^13^	*U*^23^
Cl1	0.02614 (19)	0.0226 (2)	0.01572 (18)	−0.00771 (14)	0.00426 (13)	−0.00293 (12)
Cl2	0.02389 (19)	0.01773 (19)	0.0245 (2)	0.00268 (13)	0.00429 (14)	0.00032 (13)
F1	0.0255 (5)	0.0325 (6)	0.0395 (6)	−0.0097 (4)	0.0100 (4)	0.0007 (5)
O2	0.0221 (5)	0.0199 (6)	0.0272 (6)	−0.0036 (4)	0.0059 (4)	−0.0061 (4)
O4	0.0204 (5)	0.0172 (5)	0.0271 (6)	−0.0031 (4)	0.0057 (4)	−0.0057 (4)
O6	0.0226 (5)	0.0229 (6)	0.0188 (5)	0.0006 (4)	0.0023 (4)	0.0011 (4)
N1	0.0359 (8)	0.0167 (7)	0.0321 (8)	−0.0040 (6)	0.0103 (6)	−0.0020 (6)
N3	0.0285 (7)	0.0174 (7)	0.0200 (6)	−0.0056 (5)	0.0046 (5)	−0.0023 (5)
C2	0.0147 (6)	0.0177 (7)	0.0281 (8)	−0.0015 (5)	0.0041 (6)	−0.0038 (6)
C4	0.0219 (7)	0.0151 (7)	0.0210 (7)	−0.0024 (6)	0.0043 (5)	−0.0024 (6)
C5	0.0214 (7)	0.0158 (7)	0.0171 (6)	−0.0011 (6)	0.0029 (5)	−0.0017 (5)
C6	0.0215 (7)	0.0160 (7)	0.0164 (7)	0.0001 (5)	0.0057 (5)	−0.0024 (5)
C7	0.0218 (7)	0.0165 (7)	0.0186 (7)	−0.0008 (6)	0.0063 (5)	−0.0019 (5)
C8	0.0239 (7)	0.0211 (7)	0.0204 (7)	−0.0011 (6)	0.0045 (6)	0.0014 (6)
C9	0.0219 (7)	0.0254 (8)	0.0237 (7)	−0.0007 (6)	0.0042 (6)	−0.0014 (6)
C10	0.0236 (7)	0.0207 (8)	0.0285 (8)	−0.0058 (6)	0.0093 (6)	−0.0045 (6)
C11	0.0297 (8)	0.0227 (8)	0.0269 (8)	−0.0032 (7)	0.0088 (6)	0.0052 (6)
C12	0.0233 (7)	0.0210 (8)	0.0231 (7)	−0.0001 (6)	0.0040 (6)	0.0028 (6)

### 1-[2,2-Dichloro-1-hydroxy-3-(4-methylphenyl)-3-oxopropyl]urea (I) Geometric parameters (Å, º)

**Table:**

Cl1—C5	1.7826 (14)	C4—C5	1.549 (2)
Cl2—C5	1.7923 (16)	C5—C6	1.549 (2)
F1—C10	1.3534 (18)	C6—C7	1.491 (2)
O2—C2	1.241 (2)	C7—C8	1.400 (2)
O4—H4	0.78 (3)	C7—C12	1.401 (2)
O4—C4	1.4032 (19)	C8—H8	0.9500
O6—C6	1.2103 (19)	C8—C9	1.389 (2)
N1—H1A	0.90 (3)	C9—H9	0.9500
N1—H1B	0.86 (2)	C9—C10	1.380 (2)
N1—C2	1.344 (2)	C10—C11	1.379 (2)
N3—H3	0.87 (2)	C11—H11	0.9500
N3—C2	1.366 (2)	C11—C12	1.385 (2)
N3—C4	1.431 (2)	C12—H12	0.9500
C4—H4A	1.0000		
			
C4—O4—H4	108.1 (19)	O6—C6—C5	116.71 (13)
H1A—N1—H1B	119 (2)	O6—C6—C7	121.55 (14)
C2—N1—H1A	116.4 (17)	C7—C6—C5	121.69 (13)
C2—N1—H1B	124.6 (17)	C8—C7—C6	124.60 (14)
C2—N3—H3	117.2 (17)	C8—C7—C12	119.10 (14)
C2—N3—C4	122.60 (14)	C12—C7—C6	116.26 (14)
C4—N3—H3	120.1 (17)	C7—C8—H8	119.7
O2—C2—N1	122.99 (15)	C9—C8—C7	120.56 (15)
O2—C2—N3	121.50 (15)	C9—C8—H8	119.7
N1—C2—N3	115.50 (15)	C8—C9—H9	120.9
O4—C4—N3	111.59 (13)	C10—C9—C8	118.28 (15)
O4—C4—H4A	109.0	C10—C9—H9	120.9
O4—C4—C5	106.95 (12)	F1—C10—C9	118.43 (15)
N3—C4—H4A	109.0	F1—C10—C11	118.56 (15)
N3—C4—C5	111.33 (13)	C11—C10—C9	123.01 (15)
C5—C4—H4A	109.0	C10—C11—H11	120.9
Cl1—C5—Cl2	110.35 (8)	C10—C11—C12	118.29 (15)
C4—C5—Cl1	109.49 (10)	C12—C11—H11	120.9
C4—C5—Cl2	107.43 (10)	C7—C12—H12	119.6
C6—C5—Cl1	110.36 (10)	C11—C12—C7	120.75 (15)
C6—C5—Cl2	107.22 (10)	C11—C12—H12	119.6
C6—C5—C4	111.93 (12)		
			
Cl1—C5—C6—O6	125.15 (13)	C4—N3—C2—O2	−4.3 (2)
Cl1—C5—C6—C7	−57.44 (16)	C4—N3—C2—N1	175.81 (14)
Cl2—C5—C6—O6	−114.62 (13)	C4—C5—C6—O6	2.95 (19)
Cl2—C5—C6—C7	62.78 (15)	C4—C5—C6—C7	−179.65 (13)
F1—C10—C11—C12	−179.17 (15)	C5—C6—C7—C8	−1.6 (2)
O4—C4—C5—Cl1	−56.04 (14)	C5—C6—C7—C12	−179.34 (13)
O4—C4—C5—Cl2	−175.90 (10)	C6—C7—C8—C9	−177.53 (15)
O4—C4—C5—C6	66.65 (15)	C6—C7—C12—C11	177.33 (15)
O6—C6—C7—C8	175.69 (15)	C7—C8—C9—C10	0.5 (2)
O6—C6—C7—C12	−2.1 (2)	C8—C7—C12—C11	−0.5 (2)
N3—C4—C5—Cl1	66.10 (14)	C8—C9—C10—F1	178.80 (15)
N3—C4—C5—Cl2	−53.75 (14)	C8—C9—C10—C11	−0.8 (3)
N3—C4—C5—C6	−171.20 (12)	C9—C10—C11—C12	0.4 (3)
C2—N3—C4—O4	−124.46 (15)	C10—C11—C12—C7	0.3 (3)
C2—N3—C4—C5	116.12 (15)	C12—C7—C8—C9	0.2 (2)

### 1-[2,2-Dichloro-1-hydroxy-3-(4-methylphenyl)-3-oxopropyl]urea (I) Hydrogen-bond geometry (Å, º)

**Table:**

*D*—H···*A*	*D*—H	H···*A*	*D*···*A*	*D*—H···*A*
N1—H1*A*···O4^i^	0.90 (3)	2.06 (3)	2.9488 (19)	172 (2)
N1—H1*B*···O2^ii^	0.86 (2)	2.48 (2)	3.297 (2)	158 (2)
N3—H3···O6^iii^	0.87 (2)	2.06 (2)	2.9055 (18)	167 (2)
O4—H4···O2^iv^	0.78 (3)	1.91 (3)	2.6596 (16)	161 (3)

Symmetry codes: (i) *x*, *y*+1, *z*; (ii) *x*, −*y*+5/2, *z*−1/2; (iii) *x*, −*y*+3/2, *z*−1/2; (iv) −*x*+2, −*y*+2, −*z*+1.

### 1-[2,2-Dichloro-3-(4-fluorophenyl)-1-hydroxy-3-oxopropyl]urea (II) Crystal data

**Table:**

C_11_H_12_Cl_2_N_2_O_3_	*F*(000) = 600
*M**_r_* = 291.13	*D*_x_ = 1.510 Mg m^−^^3^
Monoclinic, *P*2_1_/*c*	Cu *K*α radiation, λ = 1.54184 Å
*a* = 12.62183 (13) Å	Cell parameters from 11708 reflections
*b* = 8.77585 (9) Å	θ = 3.5–80.3°
*c* = 11.59930 (11) Å	µ = 4.60 mm^−^^1^
β = 94.8294 (9)°	*T* = 100 K
*V* = 1280.26 (2) Å^3^	Prism, colourless
*Z* = 4	0.45 × 0.35 × 0.16 mm

### 1-[2,2-Dichloro-3-(4-fluorophenyl)-1-hydroxy-3-oxopropyl]urea (II) Data collection

**Table:**

XtaLAB Synergy, Dualflex, HyPix diffractometer	2808 independent reflections
Radiation source: micro-focus sealed X-ray tube, PhotonJet (Cu) X-ray Source	2738 reflections with *I* > 2σ(*I*)
Mirror monochromator	*R*_int_ = 0.050
Detector resolution: 10.0000 pixels mm^-1^	θ_max_ = 80.7°, θ_min_ = 3.5°
ω scans	*h* = −13→16
Absorption correction: gaussian (CrysAlisPro; Rigaku OD, 2024)	*k* = −11→11
*T*_min_ = 0.277, *T*_max_ = 1.000	*l* = −14→14
17344 measured reflections	

### 1-[2,2-Dichloro-3-(4-fluorophenyl)-1-hydroxy-3-oxopropyl]urea (II) Refinement

**Table:**

Refinement on *F*^2^	Primary atom site location: dual
Least-squares matrix: full	Secondary atom site location: difference Fourier map
*R*[*F*^2^ > 2σ(*F*^2^)] = 0.037	Hydrogen site location: mixed
*wR*(*F*^2^) = 0.104	H atoms treated by a mixture of independent and constrained refinement
*S* = 1.06	*w* = 1/[σ^2^(*F*_o_^2^) + (0.0655*P*)^2^ + 0.6302*P*] where *P* = (*F*_o_^2^ + 2*F*_c_^2^)/3
2808 reflections	(Δ/σ)_max_ = 0.001
180 parameters	Δρ_max_ = 0.51 e Å^−^^3^
0 restraints	Δρ_min_ = −0.43 e Å^−^^3^

### 1-[2,2-Dichloro-3-(4-fluorophenyl)-1-hydroxy-3-oxopropyl]urea (II) Special details

**Table:**

Geometry. All esds (except the esd in the dihedral angle between two l.s. planes) are estimated using the full covariance matrix. The cell esds are taken into account individually in the estimation of esds in distances, angles and torsion angles; correlations between esds in cell parameters are only used when they are defined by crystal symmetry. An approximate (isotropic) treatment of cell esds is used for estimating esds involving l.s. planes.

### 1-[2,2-Dichloro-3-(4-fluorophenyl)-1-hydroxy-3-oxopropyl]urea (II) Fractional atomic coordinates and isotropic or equivalent isotropic displacement parameters (Å^2^)

**Table:**

	*x*	*y*	*z*	*U*_iso_*/*U*_eq_	
Cl1	0.22629 (3)	0.88241 (4)	0.40687 (3)	0.02066 (13)	
Cl2	0.24819 (3)	0.70279 (5)	0.61915 (3)	0.02309 (13)	
O2	0.02392 (10)	0.37516 (13)	0.31405 (10)	0.0221 (3)	
O4	0.03286 (9)	0.72384 (13)	0.48926 (10)	0.0192 (2)	
O6	0.27591 (10)	0.45882 (14)	0.39418 (11)	0.0259 (3)	
N1	0.03826 (12)	0.49974 (17)	0.14562 (12)	0.0212 (3)	
N3	0.08327 (11)	0.62287 (15)	0.31617 (12)	0.0180 (3)	
C2	0.04683 (12)	0.49344 (18)	0.26185 (13)	0.0181 (3)	
C4	0.10682 (12)	0.63031 (17)	0.43899 (13)	0.0176 (3)	
H4A	0.103457	0.525355	0.472057	0.021*	
C5	0.22071 (13)	0.69474 (17)	0.46569 (13)	0.0185 (3)	
C6	0.30485 (13)	0.58690 (19)	0.41747 (13)	0.0204 (3)	
C7	0.41441 (13)	0.6404 (2)	0.40165 (14)	0.0225 (3)	
C8	0.46819 (15)	0.5634 (2)	0.31877 (16)	0.0279 (4)	
H8	0.433483	0.484415	0.274165	0.033*	
C9	0.57245 (15)	0.6024 (2)	0.30150 (17)	0.0321 (4)	
H9	0.607516	0.551826	0.243057	0.039*	
C10	0.62617 (15)	0.7138 (2)	0.36820 (18)	0.0325 (4)	
C11	0.57243 (15)	0.7900 (2)	0.45141 (18)	0.0326 (4)	
H11	0.608274	0.866470	0.497716	0.039*	
C12	0.46717 (15)	0.7555 (2)	0.46742 (16)	0.0275 (4)	
H12	0.431028	0.810101	0.523000	0.033*	
C13	0.74130 (16)	0.7514 (3)	0.3535 (2)	0.0440 (5)	
H13A	0.762750	0.702446	0.283178	0.066*	
H13B	0.786149	0.713990	0.420656	0.066*	
H13C	0.749543	0.862022	0.347188	0.066*	
H1A	0.059 (2)	0.574 (3)	0.110 (2)	0.030 (6)*	
H1B	0.0183 (19)	0.420 (3)	0.110 (2)	0.028 (6)*	
H3	0.0725 (18)	0.701 (3)	0.280 (2)	0.023 (5)*	
H4	0.014 (2)	0.683 (3)	0.543 (3)	0.034 (6)*	

### 1-[2,2-Dichloro-3-(4-fluorophenyl)-1-hydroxy-3-oxopropyl]urea (II) Atomic displacement parameters (Å^2^)

**Table:**

	*U*^11^	*U*^22^	*U*^33^	*U*^12^	*U*^13^	*U*^23^
Cl1	0.0239 (2)	0.0173 (2)	0.0211 (2)	−0.00221 (12)	0.00407 (14)	0.00018 (12)
Cl2	0.0263 (2)	0.0291 (2)	0.01385 (19)	0.00153 (14)	0.00155 (14)	−0.00215 (13)
O2	0.0313 (6)	0.0194 (6)	0.0164 (5)	−0.0060 (4)	0.0065 (4)	−0.0001 (4)
O4	0.0229 (5)	0.0207 (5)	0.0150 (5)	0.0016 (4)	0.0073 (4)	0.0021 (4)
O6	0.0270 (6)	0.0222 (6)	0.0290 (6)	0.0015 (5)	0.0057 (5)	−0.0049 (5)
N1	0.0304 (7)	0.0189 (6)	0.0144 (6)	−0.0061 (5)	0.0037 (5)	−0.0009 (5)
N3	0.0253 (6)	0.0152 (6)	0.0139 (6)	−0.0010 (5)	0.0032 (5)	0.0016 (5)
C2	0.0199 (7)	0.0179 (7)	0.0168 (7)	0.0003 (5)	0.0043 (5)	0.0001 (5)
C4	0.0217 (7)	0.0176 (7)	0.0139 (7)	−0.0012 (5)	0.0042 (5)	0.0008 (5)
C5	0.0225 (7)	0.0182 (7)	0.0150 (7)	−0.0007 (6)	0.0035 (5)	−0.0005 (5)
C6	0.0235 (7)	0.0226 (7)	0.0156 (7)	0.0027 (6)	0.0039 (5)	−0.0006 (6)
C7	0.0215 (7)	0.0258 (8)	0.0203 (7)	0.0026 (6)	0.0030 (6)	0.0021 (6)
C8	0.0277 (8)	0.0307 (9)	0.0258 (8)	0.0044 (7)	0.0061 (6)	−0.0006 (7)
C9	0.0279 (9)	0.0390 (10)	0.0308 (9)	0.0081 (8)	0.0105 (7)	0.0065 (7)
C10	0.0231 (8)	0.0377 (10)	0.0372 (10)	0.0018 (7)	0.0063 (7)	0.0162 (8)
C11	0.0269 (9)	0.0346 (10)	0.0358 (10)	−0.0041 (7)	0.0000 (7)	0.0040 (8)
C12	0.0263 (8)	0.0305 (9)	0.0260 (8)	−0.0010 (7)	0.0039 (6)	−0.0010 (7)
C13	0.0258 (10)	0.0529 (14)	0.0546 (13)	−0.0018 (9)	0.0104 (9)	0.0210 (11)

### 1-[2,2-Dichloro-3-(4-fluorophenyl)-1-hydroxy-3-oxopropyl]urea (II) Geometric parameters (Å, º)

**Table:**

Cl1—C5	1.7864 (16)	C6—C7	1.486 (2)
Cl2—C5	1.7861 (16)	C7—C8	1.397 (2)
O2—C2	1.248 (2)	C7—C12	1.400 (3)
O4—C4	1.4060 (18)	C8—H8	0.9500
O4—H4	0.78 (3)	C8—C9	1.390 (3)
O6—C6	1.205 (2)	C9—H9	0.9500
N1—C2	1.345 (2)	C9—C10	1.388 (3)
N1—H1A	0.82 (3)	C10—C11	1.396 (3)
N1—H1B	0.84 (3)	C10—C13	1.514 (2)
N3—C2	1.360 (2)	C11—H11	0.9500
N3—C4	1.432 (2)	C11—C12	1.390 (3)
N3—H3	0.81 (3)	C12—H12	0.9500
C4—H4A	1.0000	C13—H13A	0.9800
C4—C5	1.551 (2)	C13—H13B	0.9800
C5—C6	1.561 (2)	C13—H13C	0.9800
			
C4—O4—H4	109 (2)	C8—C7—C6	116.30 (16)
C2—N1—H1A	121.9 (18)	C8—C7—C12	119.18 (16)
C2—N1—H1B	117.0 (16)	C12—C7—C6	124.44 (15)
H1A—N1—H1B	121 (2)	C7—C8—H8	120.0
C2—N3—C4	122.18 (13)	C9—C8—C7	120.07 (18)
C2—N3—H3	115.9 (17)	C9—C8—H8	120.0
C4—N3—H3	118.9 (17)	C8—C9—H9	119.4
O2—C2—N1	121.23 (15)	C10—C9—C8	121.10 (18)
O2—C2—N3	123.58 (14)	C10—C9—H9	119.4
N1—C2—N3	115.19 (14)	C9—C10—C11	118.69 (17)
O4—C4—N3	110.47 (13)	C9—C10—C13	121.3 (2)
O4—C4—H4A	109.1	C11—C10—C13	120.0 (2)
O4—C4—C5	109.93 (12)	C10—C11—H11	119.6
N3—C4—H4A	109.1	C12—C11—C10	120.89 (19)
N3—C4—C5	109.05 (12)	C12—C11—H11	119.6
C5—C4—H4A	109.1	C7—C12—H12	120.0
Cl2—C5—Cl1	109.48 (8)	C11—C12—C7	120.02 (17)
C4—C5—Cl1	108.99 (11)	C11—C12—H12	120.0
C4—C5—Cl2	108.20 (10)	C10—C13—H13A	109.5
C4—C5—C6	110.76 (13)	C10—C13—H13B	109.5
C6—C5—Cl1	111.85 (11)	C10—C13—H13C	109.5
C6—C5—Cl2	107.47 (11)	H13A—C13—H13B	109.5
O6—C6—C5	116.31 (14)	H13A—C13—H13C	109.5
O6—C6—C7	122.32 (15)	H13B—C13—H13C	109.5
C7—C6—C5	121.38 (14)		
			
Cl1—C5—C6—O6	−139.23 (13)	C4—N3—C2—N1	−173.78 (14)
Cl1—C5—C6—C7	40.85 (18)	C4—C5—C6—O6	−17.42 (19)
Cl2—C5—C6—O6	100.57 (15)	C4—C5—C6—C7	162.65 (14)
Cl2—C5—C6—C7	−79.35 (16)	C5—C6—C7—C8	−155.04 (15)
O4—C4—C5—Cl1	−60.19 (14)	C5—C6—C7—C12	28.2 (2)
O4—C4—C5—Cl2	58.79 (14)	C6—C7—C8—C9	−177.58 (17)
O4—C4—C5—C6	176.34 (12)	C6—C7—C12—C11	175.43 (17)
O6—C6—C7—C8	25.0 (2)	C7—C8—C9—C10	2.2 (3)
O6—C6—C7—C12	−151.71 (18)	C8—C7—C12—C11	−1.2 (3)
N3—C4—C5—Cl1	61.06 (14)	C8—C9—C10—C11	−1.9 (3)
N3—C4—C5—Cl2	−179.97 (10)	C8—C9—C10—C13	177.11 (18)
N3—C4—C5—C6	−62.41 (16)	C9—C10—C11—C12	−0.1 (3)
C2—N3—C4—O4	−110.90 (16)	C10—C11—C12—C7	1.6 (3)
C2—N3—C4—C5	128.18 (15)	C12—C7—C8—C9	−0.6 (3)
C4—N3—C2—O2	5.2 (2)	C13—C10—C11—C12	−179.03 (19)

### 1-[2,2-Dichloro-3-(4-fluorophenyl)-1-hydroxy-3-oxopropyl]urea (II) Hydrogen-bond geometry (Å, º)

**Table:**

*D*—H···*A*	*D*—H	H···*A*	*D*···*A*	*D*—H···*A*
N1—H1*A*···O4^i^	0.82 (3)	2.27 (3)	3.0261 (19)	153 (2)
N1—H1*B*···O4^ii^	0.84 (3)	2.14 (3)	2.9798 (18)	177 (2)
N3—H3···O2^iii^	0.81 (3)	2.19 (3)	2.9445 (18)	156 (2)
O4—H4···O2^iv^	0.78 (3)	1.83 (3)	2.5979 (16)	168 (3)

Symmetry codes: (i) *x*, −*y*+3/2, *z*−1/2; (ii) −*x*, *y*−1/2, −*z*+1/2; (iii) −*x*, *y*+1/2, −*z*+1/2; (iv) −*x*, −*y*+1, −*z*+1.
